# Long noncoding RNA *TUG1* contributes to cerebral ischaemia/reperfusion injury by sponging mir‐145 to up‐regulate *AQP4* expression

**DOI:** 10.1111/jcmm.14712

**Published:** 2019-11-11

**Authors:** Weifeng Shan, Wei Chen, Xian Zhao, Aijie Pei, Manli Chen, Yang Yu, Yueying Zheng, Shengmei Zhu

**Affiliations:** ^1^ Department of Anesthesiology The 1st Affiliated Hospital School of Medicine Zhejiang University Hangzhou China; ^2^ Cancer Institute of Integrated traditional Chinese and Western Medicine Zhejiang Academy of Traditional Chinese Medicine Tongde hospital of Zhejiang Province Hangzhou China

**Keywords:** Aquaporin‐4, cerebral ischaemia, lncTUG1, miR‐145, reperfusion injury

## Abstract

Emerging studies have shown that long noncoding RNA (lncRNA) *TUG1* (taurine‐up‐regulated gene 1) plays critical roles in multiple biological processes. However, the expression and function of lncRNA *TUG1* in cerebral ischaemia/reperfusion injury have not been reported yet. In this study, we found that LncRNA *TUG1* expression was significantly up‐regulated in cultured MA‐C cells exposed to OGD/R injury, while similar results were also observed in MCAO model. Mechanistically, knockdown of *TUG1* decreased lactate dehydrogenase levels and the ratio of apoptotic cells and promoted cell survival in vitro. Moreover, knockdown of *TUG1* decreased *AQP4* (encoding aquaporin 4) expression to attenuate OGD/R injury. *TUG1* could interact directly with miR‐145, and down‐regulation of miR‐145 could efficiently reverse the function of *TUG1* siRNA on *AQP4* expression. Finally, the TUG1 shRNA reduced the infarction area and cell apoptosis in I/R mouse brains in vivo. In summary, our results suggested that lncRNA *TUG1* may function as a competing endogenous RNA (ceRNA) for miR‐145 to induce cell damage, possibly providing a new therapeutic target in cerebral ischaemia/reperfusion injury.

## 1 INTRODUCTION

Ischaemia/reperfusion (I/R) is considered important for the recovery of ischaemic brain injury and also limits subsequent infarction development.^1^ However, ischaemia/reperfusion induces apoptosis, inflammation and the overproduction of reactive oxygen species (ROS), thus leading to neuronal injury.^2^ Thrombolytic therapy has been the only accepted treatment for cerebral I/R injury in clinics.^2^, ^3^ However, this therapy inevitably leads to ischaemia/reperfusion injury (IRI) and its treatment effect is limited. Accumulating evidence shows that apoptosis and death of nerve cells after I/R are the main causes of aggravating brain injury.^4^, ^5^ Therefore, it is important to develop novel therapeutic strategies to treat cerebral IRI.

In the human genome, only 2% of the DNA is translated into proteins, suggesting that the majority of the genome remains either un‐transcribed or (more often) transcribed into non–protein‐coding RNAs.^6^ According to their length, non–protein‐coding RNAs are divided into two groups, small and long noncoding RNAs. Long noncoding RNAs (lncRNAs) are longer than 200 nucleotides. They cannot be translated into proteins; therefore, initially, lncRNAs were considered as non‐functional junk. However, currently, they are thought to participate in the regulation of protein expression.^7^, ^8^ Taurine‐up‐regulated gene 1 (*TUG1*), a 7.1‐kb gene located at chr22q12.2, was initially identified as a transcript up‐regulated in retinal cells treated with taurine.^9^ Increasing evidence suggests that *TUG1* is involved in the development of a variety of cancers, including laryngeal squamous cell carcinoma and ovarian cancer.^10^, ^11^ It has been also reported that *TUG1* was up‐regulated in the brain of middle cerebral artery occlusion (MCAO) and oxygen‐glucose deprivation (OGD)‐treated SH‐SY5Y cells, indicating the therapeutic potential of *TUG1* in I/R.^12^ However, there is limited knowledge on how *TUG1* acts at the molecular level and its exact role in I/R.

Recently, many studies have demonstrated that lncRNAs regulate gene expression at various levels, including chromatin modification, transcriptional regulation and post‐transcriptional regulation.^13^ LncRNAs can act as competing endogenous RNAs (ceRNAs) to reduce the concentrations of microRNAs (miRNAs), resulting in inhibition of miRNA functions in cells.^14^ For example, lncRNA *TUG1* promotes cell proliferation and metastasis by negatively regulating miR‐300 in gallbladder carcinoma.^15^ In addition, *TUG1* alleviates extracellular matrix accumulation via mediating microRNA‐377 targeting of PPAR in diabetic nephropathy.^16^ These results prompted us to explore the mechanism of *TUG1* in cerebral I/R.

Aquaporin‐4 (AQP4) is mainly expressed in astrocyte, particularly on the end of astrocyte at the blood‐brain barrier.^17^ Early study has shown that AQP4 inhibition in mice reduces brain oedema after ischaemic stroke.^18^ Evidence has revealed that AQP4 is involved in enhancing brain oedema and AQP4 inhibition reduced brain oedema and infarct volume after ischaemic.^19^ Our previous studies also showed that AQP4 overexpression could aggravate astrocyte ischaemic/reperfusion injury.^20^, ^21^

In the present study, we employed a MCAO model and MA‐C cell oxygen‐glucose deprivation and reperfusion (OGD/R) model to determine whether *TUG1* expression was altered in cerebral IRI. We found that *TUG1* was significantly up‐regulated after OGD/R treatment and in the MCAO model. Subsequent functional studies revealed that *TUG1* silencing attenuated the OGD/R injury both in vitro and in vivo. Mechanistically, *TUG1* acts as a miRNA sponge to positively modulate the expression of *AQP4* by sponging miR‐145. Therefore, our study provides new insights into the molecular function of the TUG1/miR‐145/AQP4 signalling pathway in the pathogenesis of cerebral IRI and highlights the potential of lncRNAs to act as new therapeutic targets in cerebral IRI.

## 2 MATERIALS AND METHODS

### 2.1 Primary cultures of cerebellar astrocytes

Primary mouse astrocytes were obtained from a post‐natal day (PND) 7 cerebral cortex, as previously described.^22^ Briefly, brain regions were dissected, mechanically dissociated and incubated with trypsin, followed by trituration, repeated washing and filtering. After counting, cells were plated at a density of 10^7^ cells in 75 cm^2^ tissue culture flasks pre‐coated with poly‐D‐lysine and grown in Dulbecco's modified Eagle's medium (DMEM; Gibco) containing 10% fetal bovine serum (FBS), 100 U/mL penicillin and 100 µg/mL streptomycin at 37°C in 5% CO_2_.

### 2.2 Establishment of the OGD/R model and MCAO model

To simulate an in vitro ischaemic‐like condition, MA‐C cells were subjected to OGD. The culture medium was replaced with deoxygenated glucose‐free DMEM, and cells were incubated in a hypoxic chamber containing 5% CO_2_, 1% O_2_ and 94% N_2_ for 6 hours. Subsequently, the MA‐C cells were returned to glucose‐containing DMEM under normal culture conditions for 24 or 48 hours for reoxygenation. The overall protocol is known as OGD/R treatment.

C57/BL6 male mice were used in this study. All animal experiment protocols were approved by the animal committee at Zhejiang University. Healthy adult C57BL/6 mice were injected with intraperitoneal anaesthesia (4% chloral hydrate). The left common carotid artery, internal carotid artery and external carotid artery were separated sequentially from the left lateral approach to the neck. A silicone cord was inserted from the common carotid artery to the middle cerebral artery. After 60 minutes of embolization, the cord was removed and ligated.

Mice were randomly divided into four groups: (a) Sham: threading without occlusion, followed by persistent perfusion (n = 5); (b) I/R group: 1 hour of ischaemia and 24 hours of reperfusion (n = 5); (c) I/R + NC: mice were administrated with negative control shRNA (sh‐NC; NC; 0.5 mg/kg, via intracerebroventricular injection) before ischaemia treatment (n = 5) and (d) I/R + *TUG1* shRNA: mice were administrated with TUG1 shRNA (0.5 mg/kg, via intracerebroventricular injection) before ischaemic treatment (n = 5). TUG1 shRNA or sh‐NC was injected into the right cerebral ventricle of mice. One day post‐injection, MCAO operation was established.

### 2.3 Transfection of *TUG1* siRNA, miR‐145 mimic, miR‐145 inhibitor or AQP4 siRNA

To investigate the lncRNA *TUG1*’s function in OGD/R‐treated MA‐C cells, miR‐145 mimics, inhibitors, negative control miRNA, *TUG1* siRNA, AQP4 siRNAs, or negative siRNA, or two of these treatments were transfected into cells at working concentrations [1:1 (v/v)] using Lipofectamine^™^ 2000 Transfection reagent (Invitrogen; Thermo Fisher Scientific, Inc) according to the manufacturer's protocol. After 6 hours of transfection, MA‐C cells underwent OGD/R treatment (6‐hour OGD and 24‐hour reoxygenation). The transfection efficiencies of the miRNA or siRNA in MA‐C cells were confirmed by quantitative real‐time reverse‐transcription polymerase chain reaction (qRT‐PCR) to measure miR‐145 or *AQP4* expression. The transfection efficiencies of the *AQP4* siRNA were confirmed using Western blotting analysis.

### 2.4 Real‐time polymerase chain reaction

Total RNA was isolated from cultured primary astrocytes or ischaemic hemisphere from MCAO mice using the TRIzol reagent (Invitrogen) following the manufacturer's protocol, and the RNA concentration was measured spectrophotometrically. Single‐stranded cDNA was synthesized using a cDNA synthesis kit (Takara) according to the manufacturer's procedures. RT‐PCR assays were performed using Applied Biosystems SYBR Green Mix kits (Applied Biosystems). The following primers were used:

Tug1‐FCAGTTCTTGCAACAGGTGAGC
Tug1‐RTACTTCAGTAGGGCCAGCCA
AQP4‐F GACAGACCCACAGCAAGG
AQP4‐F GCAAAGGGAGATGAGAACC
mir‐145 GTCCAGTTTTCCCAGGAATCCCT

All experiments were repeated three times, and the results were normalized to the expression of actin or U6 mRNA.

### 2.5 Western blot analysis

Proteins from cultured primary astrocytes were extracted using protein lysis solution (Cell Signaling Technology, Inc) containing protease inhibitors (Sigma‑Aldrich; Merck KGaA) and centrifuged at 12 000 × *g* for 5 minutes at 4°C. The supernatant was collected and quantified using the bicinchoninic acid method. Equivalent amounts of proteins (40 μg/lane) were separated on 10% SDS‐PAGE gels and then transferred to polyvinylidene fluoride (PVDF) membranes (Millipore). The membranes were blocked with 5% non‐fat milk in TBST buffer for 1 hour and then incubated overnight with the following primary antibodies: anti‐AQP4 (ab46182, Abcam) and anti‐β‐actin (66009‐1, Proteintech).

After being washed twice with TBST, the membranes were incubated with a horseradish peroxidase‐conjugated secondary antibody, anti‐rabbit‐HPR (7074, Cell Signaling Technology [CST]) or anti‐mouse‐HPR (7076, CST) at 1:2000 dilution. Specific bands were visualized using an enhanced chemiluminescence detection kit.

### 2.6 Lactate dehydrogenase assay

The death of MA‐C cells was evaluated using a cytotoxicity lactate dehydrogenase (LDH) assay kit (DOJINDO) according to the manufacturer's protocol. Briefly, MA‐C cells were cultured in 6‐well plates, following OGD/R treatment and transfection. MA‐C cells were collected, resuspended and cultured in 96‐well plates in 37°C in CO_2_ for the appropriate time. Then, 10 µL of Lysis Buffer was added and the cells were cultured at 37°C in CO_2_ for 30 minutes. Working solution was then added at 100 µL per well, and the samples were cultured at room temperature in the dark. Stop solution (50 µL) was then added to each well, and LDH levels in the culture supernatant were analysed immediately by measuring the absorbance at 490 nm using a microplate reader. LDH levels in the control group were expressed as 100%, and the levels in other groups were normalized to this value.

### 2.7 Cell health assay

Live or dead MA‐C cells were evaluated using a Calcein‐AM/PI Double Stain Kit (DOJINDO) according to the manufacturer's protocol. Briefly, MA‐C cells were cultured in 6‐well plates, following OGD/R treatment and transfection. MA‐C cells were collected, resuspended and cultured in 96‐well plates in 37°C CO_2_ for the appropriate time. Calcein‐AM reserve solution (10 µL) and 15 µL of propidium iodide (PI) reserve solution were added to 5 mL of serum‐free medium to prepare the dyeing solution. The final concentration of Calcein‐AM was 2 µmol/L, and the final concentration of PI was 4 µmol/L. Dyeing solution (100 µL) was added each well. The cells were then cultured 37°C for 30 minutes before washing twice with phosphate‐buffered saline (PBS). Live cells were detected by excitation at 490 nm, and dead cells were detected by excitation at 545 nm using a microplate reader.

### 2.8 TdT‐mediated biotin‐16‐dUTP nick‐end labelling assay

The TdT‐mediated biotin‐16‐dUTP nick‐end labelling (TUNEL) assay was performed using a One‐Step TUNEL Apoptosis Assay kit (Roche) to detect apoptotic cells in the mouse brain. In brief, 4‐μm‐thick paraffin sections were deparaffinized, hydrated, treated with proteinase K for 20 minutes and subsequently incubated with a mixture of a fluorescently labelled solution of dUTP and the TdT enzyme at 37°C for 1 hour in a humidified atmosphere. As a positive control, sections were incubated with DNase I for 10 minutes at room temperature (25°C) before the fluorescent labelling procedure. Negative controls were incubated with dUTP for 10 minutes at room temperature (25°C). Subsequently, the samples were treated with diaminobenzidine, counterstained with haematoxylin (to identify the cell nuclei). The cells were then dehydrated in a gradient ethanol series, vitrified with dimethylbenzene and mounted with neutral balsam.

### 2.9 2,3,5‐Triphenyltetrazolium chloride measurement

Animals were anaesthetized with 4% chloral hydrate, and brain tissues were sectioned coronally and consecutively at 2‐mm thick. Coronal brain slices were incubated in 1% 2,3,5‐Triphenyltetrazolium chloride (TTC) buffer, at 37°C for 20 minutes. After incubation, the brain slices were fixed in 4% paraformaldehyde for 24 hours and then photographed. The red regions represented non‐infarcted tissue regions, and pale white regions are infarcted tissue regions.

### 2.10 Neurological function assessment

The severity of the injury of treated mice was assessed using a modified Bederson score as previously described.^23^ The modified Bederson scores were as follows: 0, no deficit; mice with score 1, lost forelimb flexion; 2, as for 1, but plus decreased resistance to a lateral push; 3, indicated unidirectional circling; mice with score 4 displayed longitudinal spinning or seizure activity; mice with score 5 showed no movement.

#### Luciferase reporter assay

293T cells were seeded in a 24‐well plate. After 24 hours, miR‐145‐5p mimics or miR‐NC was cotransfected with TUG1‐wt (wild type) or TUG1‐mut (mutant) into 293T cells using Lipofectamine 3000 (Invitrogen), dual‐luciferase reporter assay system (Promega) were applied at 48 hours after transfection according to the manufacturer's instructions.

### 2.11 Apoptosis assay

Apoptosis was detected using an Annexin V‐FITC Apoptosis Detection Kit (DOJINDO) according to the manufacturer's protocol. Briefly, MA‐C cells were cultured in 6‐well plates, following OGD/R treatment and transfection. The cells were collected, resuspended with 195 μL Annexin V‐FITC binding solution. Annexin V‐FITC (5 μL) was then added, mixed gently and incubated for 10 minutes at room temperature (20‐25°C) in the dark. The cells were centrifuged, resuspended in 190 μL of Annexin V‐FITC binding solution. Then, 10 μL of PI solution was added, mixed well and placed in ice bath. Data were acquired using a BD FACSCalibur flow cytometer and analysed using BD Cell Quest software.

### 2.12 Immunofluorescence

Immunofluorescence was used to identify astrocytes. Cells were washed with cold PBS, fixed in 4% paraformaldehyde for 15 minutes, blocked with 5% serum at 37°C for 60 minutes and incubated with a 1:300 dilution of anti‐glial fibrillary acidic protein (GFAP) antibodies (#3670; CST) at 4°C overnight. After washing with PBS three times, the cells were incubated with a 1:100 dilution of a secondary antibody (#4409; CST) at 37°C for 2 hours. 2‐(4‐amidinophenyl)‐1H‐indole‐6‐carboxamidine (DAPI; Sigma‐Aldrich; Merck KGaA) was used for nuclear staining at 37°C for 2 minutes, and the cells were then washed three times with PBS and observed using an inverted fluorescence microscope (Olympus Corp.).

### 2.13 Statistical analysis

Statistical analysis was performed using GraphPad Prism 5.0 software (GraphPad Software). Data were presented as a mean ± standard deviation (SD). To compare the difference between two groups or multiple groups, a t test and one‐way analysis of variance (ANOVA) were performed, respectively. A difference was considered significant at *P* < .05.

## 3 RESULTS

### 3.1 *TUG1* is highly expressed after OGD/R treatment

**Figure 1:**
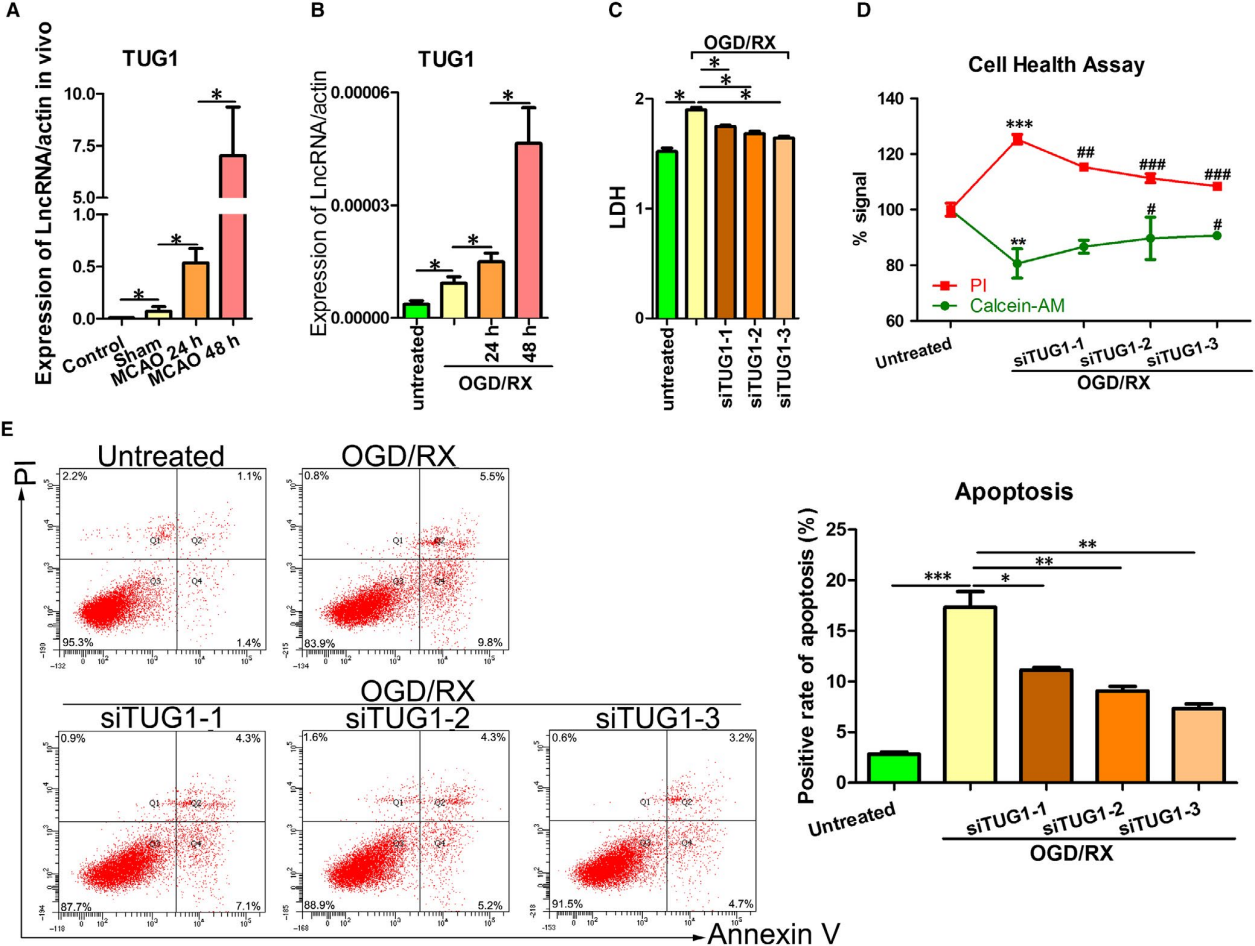
*TUG1* was up‐regulated in MA‐C cells cultured under OGD/R conditions. A, QRT‐PCR showing *TUG1* levels in MA‐C cells at 24 or 48 h post‐reoxygenation. B, *TUG1* levels were measured using qRT‐PCR at 24 h/48 h post‐reperfusion after MCAO was established. C, LDH leakage was measured using LDH assays after *TUG1* knockdown under OGD‐R conditions (6‐h OGD and 24‐h reoxygenation). D, Cell health was measured using cell health assays after *TUG1* knockdown under OGD‐R conditions (6‐h OGD and 24‐h reoxygenation). E, Flow cytometry assays were performed to show the cell apoptosis after transfection with short interfering RNAs siTUG1‐1, siTUG1‐2 and siTUG1‐3 after 6‐h OGD/24‐h reoxygenation treatments. MCAO, middle carotid artery occlusion; OGD/R, oxygen‐glucose deprivation and reperfusion; qRT‐PCR, quantitative real‐time reverse‐transcription polymerase chain reaction; TUG1, taurine‐up‐regulated gene 1

To investigate whether *TUG1* expression was altered in cerebral IRI, *TUG1* expression in MA‐C cells was measured immediately following 6 hours of OGD and during different reoxygenation times using qRT‐PCR. The morphological changes of MA‐C cells under control or OGD/RX condition were shown in Figure [Link], [Link]. High levels of TUG1 were detected in the MCAO model (ischaemia for 1 hour and during different reperfusion time periods) compared with that in the control group (*P* < .05; Figure 1A). Moreover, TUG1 levels were significantly up‐regulated under OGD/R conditions compared with that under the control conditions (*P* < .05; Figure 1B). These results suggested that the up‐regulation of lncRNA *TUG1* contributes to cerebral ischaemia injury.

Given that *TUG1* is overexpressed in MA‐C cells exposed to OGD/R treatment, we performed knockdown of *TUG1 *in vitro to investigate whether *TUG1* plays a role in IRI. Three small interfering RNA (siRNAs) targeting the coding region of *TUG1* (siTUG1‐3) were tested for their knockdown efficiency. Cells were transfected with siTUG1‐1, siTUG1‐2, siTUG1‐3 or a negative control. The efficiency of interference was verified by qRT‐PCR (Figure [Link], [Link]). The effects of *TUG1* on OGD/R were evaluated using LDH assays and cell health assays. Silencing of *TUG1* significantly suppressed LDH leakage and promoted cell survival compared with the non‐transfected ODG/R‐treated group (Figure 1C‐D). In addition, OGD/R treatment increased cell apoptosis while silencing of *TUG1* prevented OGD/R‐induced apoptosis (Figure 1E). These results suggested that OGD/R induces cell injury by up‐regulating *TUG1* expression.

### 3.2 Knockdown of *TUG1* attenuates the OGD/R injury by inhibiting the expression of AQP4

**Figure 2:**
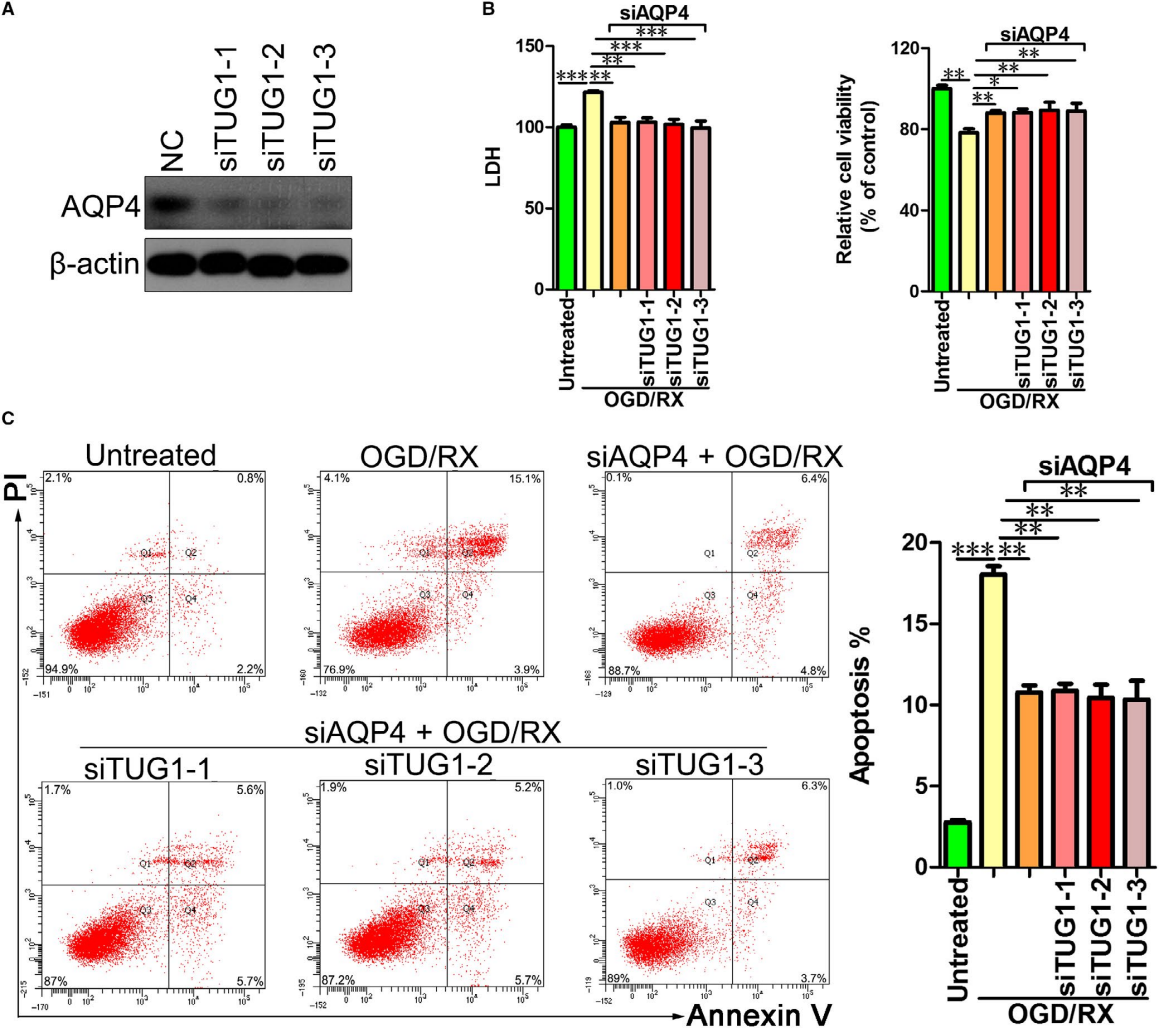
Knockdown of *TUG1* attenuates the OGD/R injury by inhibiting the expression of AQP4. A, AQP4 protein levels in MA‐C cells transfected with short interfering RNAs siTUG1‐1, siTUG1‐2 and siTUG1‐3 as assessed using by Western blotting assays. B, LDH leakage (left) and cell viability were measured using LDH and CCK8 assays, respectively. MA‐C cells were transfected with siTUG1‐1, siTUG1‐2 and siTUG1‐3 in 6‐h OGD/24‐h reoxygenation treatments. C, Flow cytometry assays were performed to show the cell apoptosis after cotransfection with AQP4 siRNA and siTUG1‐1, siTUG1‐2 and siTUG1‐3 in 6‐h OGD/24‐h reoxygenation treatments. AQP4, aquaporin 4; OGD/R, oxygen‐glucose deprivation and reperfusion; siRNA, short interfering RNA; TUG1, taurine‐up‐regulated gene 1

Our team has been studying AQP4 and brain tissue injury in the early stage and found that AQP4 plays an important role in cerebral injury in stroke.^20^, ^21^ This was confirmed in Figure S, which showed that knockdown of *AQP4* inhibits cell apoptosis and LDH leakage, and increases cell health in OGD/R‐treated MA‐C cells. Therefore, we hypothesized that *TUG1* might exert its function by regulating *AQP4* expression. To confirm whether *TUG1* regulates *AQP4*, MA‐C cells were transfected with siTUG1‐1, siTUG1‐2, siTUG1‐3 or the negative control. As expected, knockdown of *TUG1* expression significantly decreased the accumulation of AQP4 (Figure 2A). Next, to confirm whether AQP4 mediates the damage of caused by *TUG1* to MA‐C cells exposed to OGD/RX, the cells were transfected with siAQP4, exposed to OGD/R, and then transfected with control, siTUG1‐1, siTUG1‐2 and siTUG1‐3, respectively. OGD/R treatment enhanced LDH leakage and suppressed cell viability, whereas knockdown of *AQP4* significantly attenuated the OGD/R treatment‐induced damage. However, knockdown of *TUG1* did not further mitigate the damage (Figure 2B). The efficiency of siAQP4 interference was verified by qRT‐PCR (Figure [Link], [Link]). In addition, after transfection with siAQP4, knockdown of *TUG1* did not further decrease the OGD/R treatment‐induced apoptosis (Figure 2C). These results indicated that *TUG1* plays a role in cerebral IRI by regulating *AQP4* expression.

### 3.3 TUG1 directly interacts with mir‐145 to regulate AQP4 protein expression

**Figure 3:**
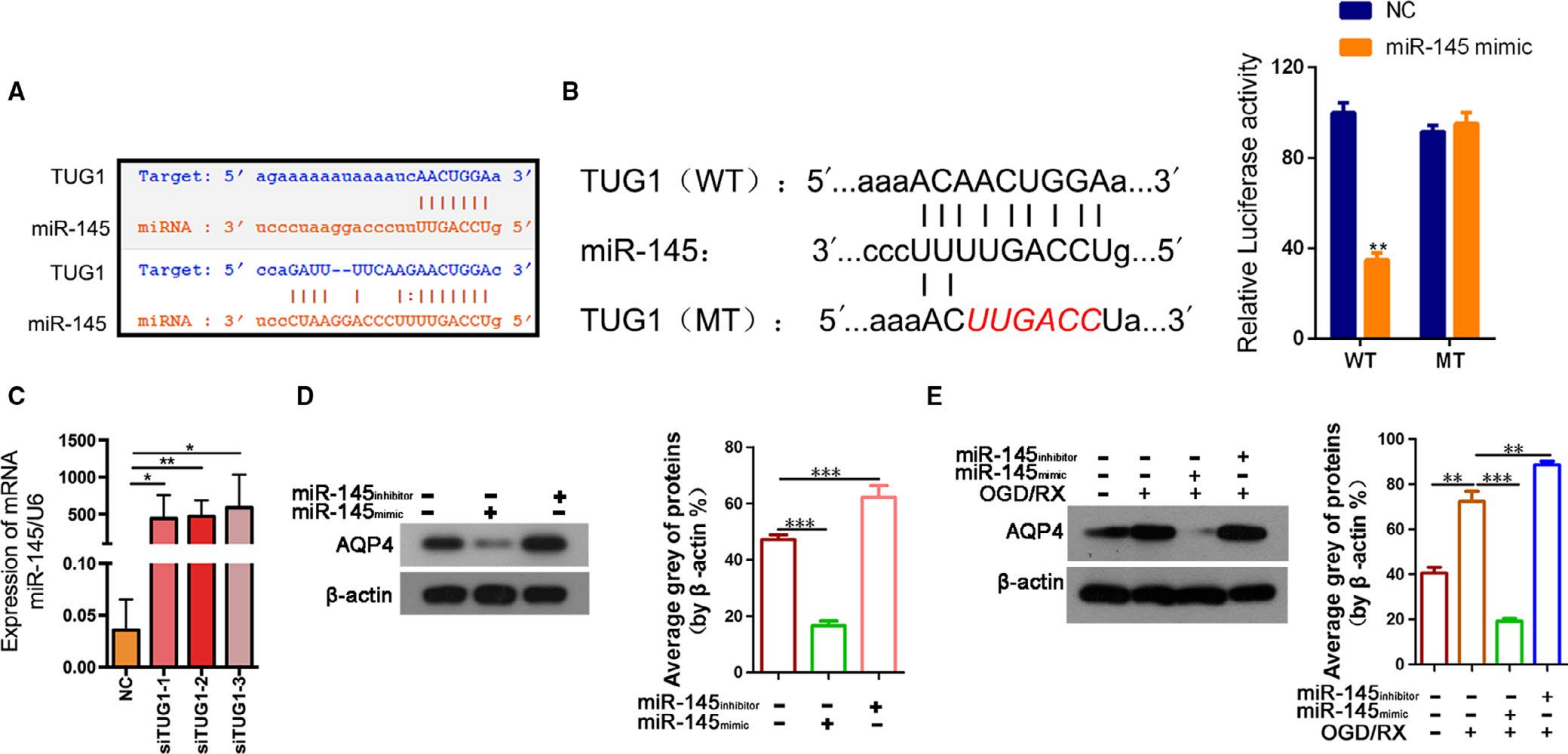
*TUG1* directly targeted miR‐145 and mir‐145 regulates the expression of *AQP4.* A, Sequence alignment of predicted mir‐145–binding sites within the TUG1. B, Schematic diagram showed the putative miR‐145‐5p–binding sites within the TUG1. The sequences of wild‐type TUG1 and mutant TUG1 were listed as well. Luciferase reporter gene assays were performed to measure the luciferase activity in 293T cells. **P < .01. C. QRT‐PCR assays were performed to analyse the expression level of miR‐145 after transfection with siTUG1‐1, siTUG1‐2 and siTUG1‐3. D, AQP4 protein level after transfection with mir‐145 mimic or inhibitor was measured by Western blotting and quantified. E, AQP4 protein level after transfection with mir‐145 mimic or inhibitor was measured in 6‐h OGD/24‐h reoxygenation treatments by Western blotting and quantified. AQP4, aquaporin 4; qRT‐PCR, quantitative real‐time reverse‐transcription polymerase chain reactionTUG1, taurine‐up‐regulated gene 1

Bioinformatic analysis of miRNA recognition sequences in *TUG1* was performed using miRcode. MiR‐145 was identified as being capable of binding to complementary sequences in TUG1 (Figure 3A). To prove the prediction, we performed luciferase reporter assay. The results showed that TUG1‐WT could significantly lower the luciferase activity in miR‐145‐5p group but had no significant effect on the luciferase activity of the miR‐NC group (Figure 3B). To determine whether the expression of miR‐145 is regulated by *TUG1*, we transfected MA‐C cells with siTUG1‐1, siTUG1‐2 and siTUG1‐3. After knockdown of *TUG1*, the mir‐145 level was significantly up‐regulated, indicating that *TUG1* negatively regulates mir‐145 (Figure 3C).

To investigate the regulatory effect of mir‐145 on *AQP4* expression, MA‐C cells were transfected with a mir‐145 mimic or mir‐145 inhibitor. The mir‐145 mimic decreased AQP4 levels, while the mir‐145 inhibitor increased AQP4 levels (Figure 3D). OGD/R treatment significantly enhanced AQP4 expression, while the mir‐145 mimic significantly suppressed the AQP4 expression induced by OGD/R treatment (Figure 3E). Taken together, these data indicated that knockdown of *TUG1* increased the expression of miR145 and inhibited the expression of *AQP4* in MA‐C cells exposed to OGD/R treatment.

### 3.4 Mir‐145 inhibitor reverses the protective effect of *TUG1* siRNA against OGD/R‐induced injury in vitro

**Figure 4:**
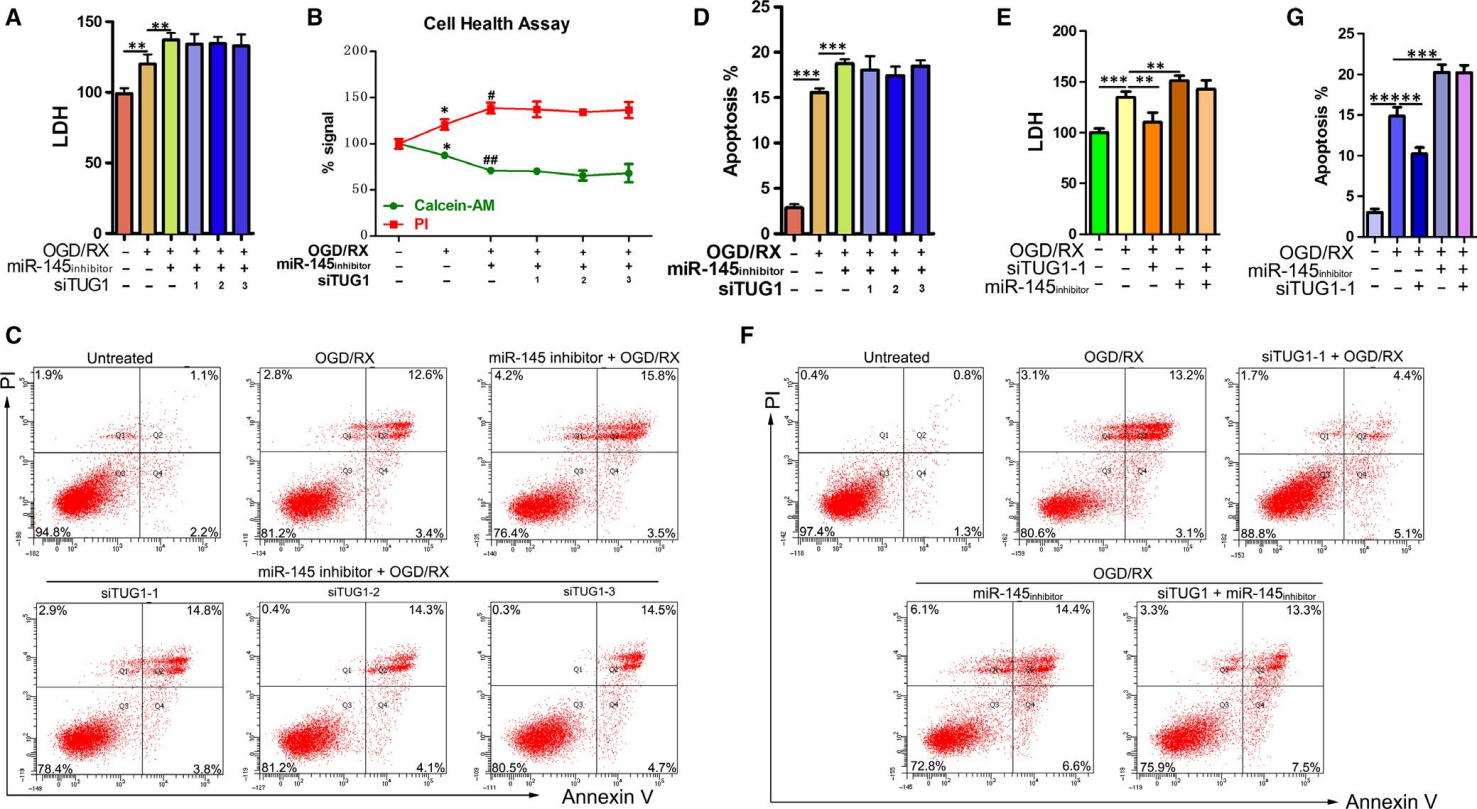
Changes in cell damage in OGD/R‐treated MA‐C cells cotransfected with the miR‐145 inhibitor and siTUG1. A, LDH leakage and B, cell health were measured using LDH assays and cell health assays. MA‐C cells were cotransfected with mir‐145 inhibitor and siTUG1‐1, siTUG1‐2 or siTUG1‐3 in 6‐h OGD/24‐h reoxygenation treatments. C‐D, Flow cytometry assays were performed to show the cell apoptosis after cotransfection with mir‐145 inhibitor and siTUG1‐1, siTUG1‐2 or siTUG1‐3 in 6‐h OGD/24‐h reoxygenation treatments. E, LDH leakage was measured using LDH assays after cotransfection with mir‐145 inhibitor and siTUG1‐1 in 6‐h OGD/24‐h reoxygenation treatments. F‐G, Flow cytometry assays were performed to show the cell apoptosis after cotransfection with mir‐145 inhibitor and siTUG1‐1 in 6‐h OGD/24‐h reoxygenation treatments. OGD/R, oxygen‐glucose deprivation and reperfusion; TUG1, taurine‐up‐regulated gene 1

Considering the inhibitory action of *TUG1* on miR‐145 levels in MA‐C cells, we further explored the role of miR‐145 in the protective effects of the *TUG1* siRNA against OGD/R‐induced injury. Astrocytes were pretreated with the miR‐145 inhibitor, transfected with untreated and siTUG1‐1, siTUG1‐2 or siTUG1‐3, and then exposed to OGD/R. LDH, cell health assays and cell apoptosis assays were performed. OGD/R treatment increased LDH leakage and decreased cell health, indicating that OGD/R treatment induces cell damage. Transfection of the miR‐145 inhibitor further aggravated cell damage, while cotransfection of miR‐145 inhibitor + si‐TUG1 did not abolished the effects induced by the miR‐145 inhibitor (Figure 4A‐B). The efficiency of mir‐145 inhibitor transfection was verified by qRT‐PCR (Figure [Link], [Link]). In line with cell apoptosis, OGD/R treatment increased cell apoptosis compared with that in the untreated group. Transfection of the miR‐145 inhibitor further enhanced cell apoptosis, while cotransfection of miR‐145 inhibitor + si‐TUG1 did not decrease cell apoptosis, suggesting that the protective effect of *TUG1* siRNA had disappeared (Figure 4C‐D).

Next, siTUG1‐1 was chosen for further experiments. Astrocytes were pretreated with siTUG1‐1, transfected separately with untreated and mir‐145 inhibitor, and then exposed to OGD/R *TUG1* knockdown significantly reduced LDH leakage, whereas cotransfection of the miR‐145 inhibitor + si‐TUG1 did not reduce LDH leakage (Figure 4E). In addition, apoptosis assays showed that *TUG1* knockdown significantly decreased cell apoptosis under OGD/R treatment, while cotransfection of the miR‐145 inhibitor + si‐TUG1 did not decrease cell apoptosis (Figure 4F‐G). Collectively, these results demonstrated the TUG1 siRNA protects against cerebral IRI by up‐regulating mir‐145 levels.

### 3.5 In vivo, TUG1 shRNA reduces the infarction area and cell apoptosis in I/R mice brains

**Figure 5:**
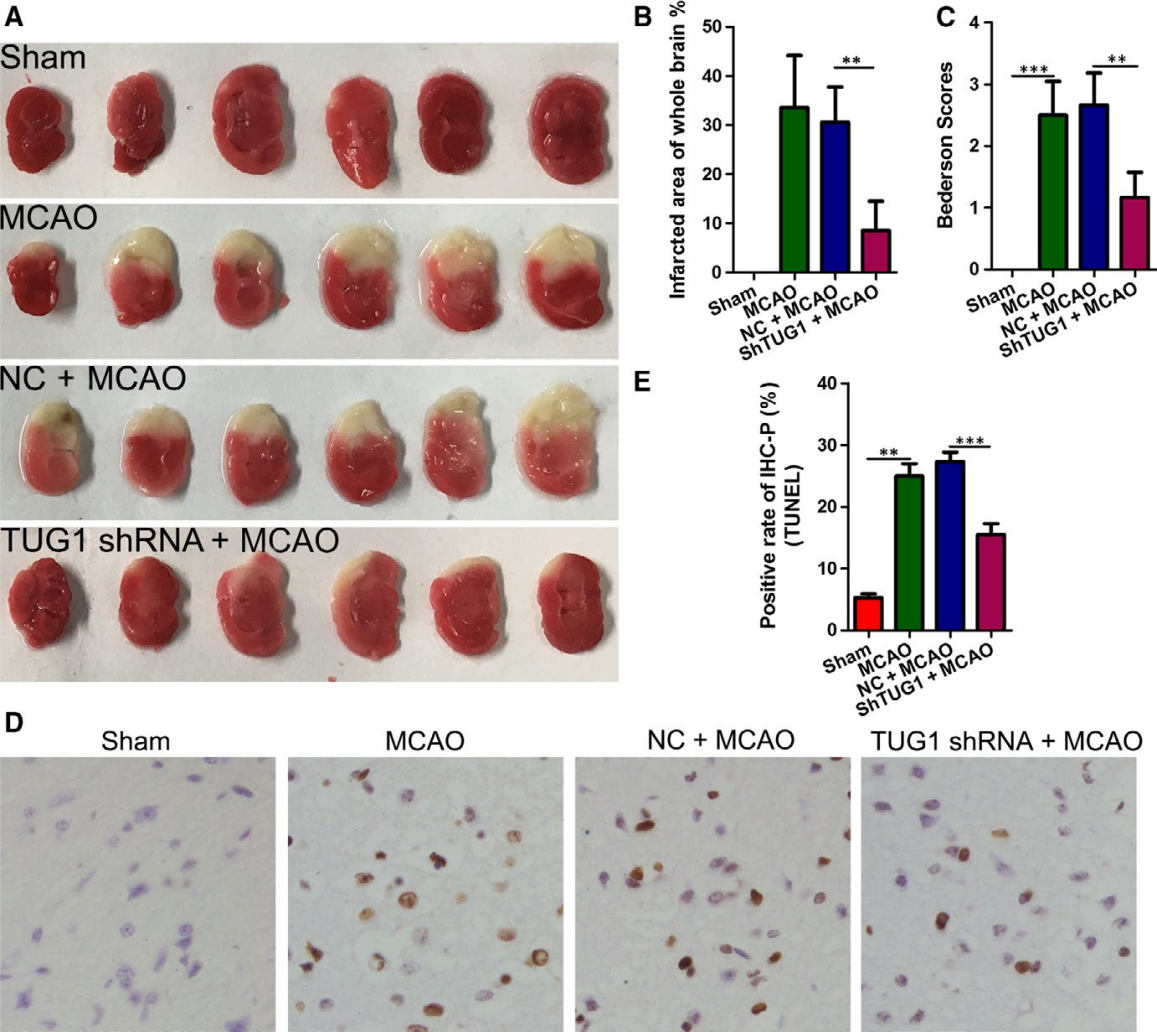
*TUG1* shRNA attenuated the infarction area and decreased cell apoptosis in I/R mouse brains. A‐B, Representative coronal sections of TTC staining after MCAO. The relative infarct area percentage was evaluated by observing the unstained infarcted tissue zone (white) and the stained normal tissue zone (red). C, Bederson scores of mice treated with or without *TUG1* shRNA after MCAO. D‐E, Cell apoptosis was examined using a TdT‐mediated biotin‐16‐dUTP nick‐end labelling (TUNEL) assay (magnification, ×400; **P < .01, ***P < .001 *vs*. sham). MCAO, middle carotid artery occlusion; shRNA, short hairpin RNA; TTC, 2,3,5‐Triphenyltetrazolium chloride; TUG1, taurine‐up‐regulated gene 1

To demonstrate the protective role of *TUG1* against brain I/R injury, we first established the MCAO model in mice and determined the effect of *TUG1* on infarction area using TTC staining, with Sham, MCAO, MCAO + NC and MCAO + TUG1 shRNA groups. As shown in Figure 5A, the infarct region was observed in the brain of both MCAO and MCAO + NC groups. However, the infarct area was significantly reduced in the MCAO group + TUG1 shRNA group (Figure 5B). Meanwhile, we used the Bederson score to rate the functional neurological deficits in MCAO mice after transfection with or without *TUG1* shRNA. The results showed that the Bederson score decreased significantly after *TUG1* knockdown (Figure 5C). Moreover, TUNEL analysis revealed that *TUG1* knockdown could significantly decrease the rate of apoptosis in MCAO mice (Figure 5D). Our data indicated that *TUG1* knockdown effectively protects against brain tissue damage in MCAO model mice.

## 4 DISCUSSION

Although evidence has demonstrated the ectopic expression of lncRNA *TUG1* in many cancers,^10^, ^11^, ^24^ its expression and biological role in cerebral IRI remain uncharacterized. Our study confirmed that *TUG1* expression was significantly higher after OGD/R treatment compared with that in the control group. High *TUG1* expression was also found in MCAO model mice compared with the control group. Moreover, shRNA‐mediated silencing of *TUG1* reduced the infarction area and cell apoptosis in I/R mouse brains. Together, these data support the concept that *TUG1* is associated with cerebral IRI and functions as a positive regulator in IRI progression. A new mechanism has been proposed in which lncRNA *H19* induces cerebral ischaemia‐reperfusion injury 25 and more recently, Weixin et al 5 demonstrated that lncRNA *MALAT1* inhibits apoptosis induced by oxygen‐glucose deprivation and reoxygenation in human brain microvascular endothelial cells. From these studies, a broader understanding of the mechanisms of action of more lncRNAs may further the development of new therapeutic strategies for cerebral IRI.

Aquaporin‐4 (AQP4), a member of the water channel family, is mainly expressed in astrocytes, ependymal cells, soft meninges, choroid plexus and the nucleus of the lower mound in the brain.^17^, ^26^ Increasing evidence indicates that AQP4 plays an important role in cerebral ischaemia/reperfusion. Combination therapy of Emodin and GRb1 had a neuroprotective effect via the down‐regulation of AQP4 in cerebral ischaemia/reperfusion rats.^27^ *AQP4*‐null mice are protected from cellular (cytotoxic) brain oedema produced by water intoxication, brain ischaemia or meningitis.^28^ Our previous studies showed AQP4 plays a role in cerebral ischaemic injury.^20^, ^21^ MiR‐29a ameliorated ischaemic injury of astrocytes in vitro by targeting AQP4,^20^ and up‐regulation of miR‐130b protects against cerebral ischaemic injury also by targeting AQP4.^21^ Therefore, we hypothesized that *TUG1* exerts its function by regulating AQP4. First, knockdown of *TUG1* decreased the accumulation of the AQP4 protein. Second, *APQ4* silencing alleviated cellular damage. However, knockdown of *TUG1* did not further mitigate the damage. Third, after transfection with siAQP4, knockdown of *TUG1* did not further decrease the OGD/R treatment‐induced apoptosis. The above data not only supported a pro‐apoptosis role of AQP4 in cerebral ischaemic injury progression, but also revealed a novel mechanism by which an lncRNA induces cell damage by regulating *AQP4* expression.

The mechanisms of action of lncRNAs include both transcriptional and post‐transcriptional regulation. Recently, a new regulatory mechanism in which lncRNAs acting as ceRNAs to sponge miRNAs, thus participating in post‐transcriptional processing, has been identified.^29^ The miRNA sponge function of *TUG1* was supported by the evidence generated in the present study. First, we predicted the interaction between *TUG1* and miR‐145 using bioinformatic analysis and found that *TUG1* indeed contains a target site of miR‐145. Second, after knockdown of *TUG1*, the mir‐145 level was significantly up‐regulated, indicating that *TUG1* negatively regulates mir‐145. Third, down‐regulation of miR‐145 level efficiently reversed the protective effect induced by the *TUG1* siRNA. These results confirmed that TUG1 induces cell damage by regulating mir‐145.

After confirming the positive relationship between *TUG1* and AQP4, as well as the negative correlation between *TUG1* and mir‐145 during cerebral I/R injury, we also identified the downstream target of mir‐145 in this mechanism. Overexpression of miR‐145 via the mir‐145 mimic decreased AQP4 accumulation, whereas down‐regulation of mir‐145 via the mir‐145 inhibitor increased AQP4 levels. In addition, the mir‐145 mimic reversed the AQP4 expression in the presence of OGD/R. These analyses showed that *TUG1* sponges miR‐145 to induce cell damage by up‐regulating AQP4. In fact, previous studies have shown that individual protein‐coding genes can be regulated by multiple non–protein‐coding RNAs, while one non–protein‐coding RNA can regulate multiple protein‐coding genes.^30^, ^31^ Thus, whether AQP4 could be regulated by other sponging lncRNAs, and whether *TUG1* could function as a sponge lncRNA to affect expressions of other key regulators in cerebral IRI, requires further investigation.

## 5 CONCLUSION

In the present study, we identified *TUG1* as a novel positive regulator in the pathogenesis of cerebral IRI. Moreover, our findings shed light on the interaction between *TUG1* and miR‐145 in IRI, and revealed that *TUG1* positively regulates post‐transcriptional expression of *AQP4* by sponging miR‐145 in cerebral IRI. Knockdown of *TUG1* via siRNA decreased *AQP4* expression, providing a novel therapeutic target for cerebral IRI.

## COMPETING INTERESTS

The authors declare that they have no competing interests.

## AUTHORS CONTRIBUTIONS

ZSM and ZYY conceived the research idea; SWF and CW performed the experiments; PAJ, CML and YY analysed the data; ZX wrote the manuscript. All authors read and approved the final version of the manuscript.

## ETHICAL APPROVAL AND CONSENT TO PARTICIPATE

All animal experiments were approved by the First Affiliated Zhejiang Hospital, Zhejiang University of Medical Ethics Committee, the Medical Faculty Ethics Committee of the First Affiliated Zhejiang Hospital, Zhejiang University in accordance with the National Institutes of Health Guide for Care and Use of Laboratory Animals (NIH Publications, No. 8023, revised 1978).

## Data Availability

All data generated or analysed during this study are included in this published article.

## References

[1] Huang Z , Lu L , Jiang T , et al. miR‐29b affects neurocyte apoptosis by targeting MCL‐1 during cerebral ischemia/reperfusion injury. Exp Ther Med. 2018;16:3399‐3404. 30233687 10.3892/etm.2018.6622 PMC6143871

[2] Duan Q , Sun W , Yuan H , Mu X . MicroRNA‐135b‐5p prevents oxygen‐glucose deprivation and reoxygenation‐induced neuronal injury through regulation of the GSK‐3beta/Nrf2/ARE signaling pathway. Arch Med Sci. 2018;14:735‐744. 30002689 10.5114/aoms.2017.71076 PMC6040137

[3] Wang SH , Ma F , Tang ZH , et al. Long non‐coding RNA H19 regulates FOXM1 expression by competitively binding endogenous miR‐342‐3p in gallbladder cancer. J Exp Clin Cancer Res. 2016;35:160. 27716361 10.1186/s13046-016-0436-6 PMC5048611

[4] Cao L , Zhang Y , Zhang S , et al. MicroRNA‐29b alleviates oxygen and glucose deprivation/reperfusion‐induced injury via inhibition of the p53‐dependent apoptosis pathway in N2a neuroblastoma cells. Exp Ther Med. 2018;15:67‐74. 29399057 10.3892/etm.2017.5410 PMC5766061

[5] Xin JW , Jiang YG . Long noncoding RNA MALAT1 inhibits apoptosis induced by oxygen‐glucose deprivation and reoxygenation in human brain microvascular endothelial cells. Exp Ther Med. 2017;13:1225‐1234. 28413461 10.3892/etm.2017.4095 PMC5377418

[6] ENCODE Project Consortium . An integrated encyclopedia of DNA elements in the human genome. Nature. 2012;489:57‐74. 22955616 10.1038/nature11247 PMC3439153

[7] Zhan Y , Zang H , Feng J , Lu J , Chen L , Fan S . Long non‐coding RNAs associated with non‐small cell lung cancer. Oncotarget. 2017;8:69174‐69184. 28978188 10.18632/oncotarget.20088 PMC5620328

[8] Mercer TR , Mattick JS . Structure and function of long noncoding RNAs in epigenetic regulation. Nat Struct Mol Biol. 2013;20:300‐307. 23463315 10.1038/nsmb.2480

[9] Young TL , Matsuda T , Cepko CL . The noncoding RNA taurine upregulated gene 1 is required for differentiation of the murine retina. Curr Biol. 2005;15:501‐512. 15797018 10.1016/j.cub.2005.02.027

[10] Liu S , Liu Y , Lu Q , Zhou X , Chen L , Liang W . The lncRNA TUG1 promotes epithelial ovarian cancer cell proliferation and invasion via the WNT/beta‐catenin pathway. Onco Targets Ther. 2018;11:6845‐6851. 30349317 10.2147/OTT.S167900 PMC6190633

[11] Zhang Z , Wang X , Cao S , et al. The long noncoding RNA TUG1 promotes laryngeal cancer proliferation and migration. Cell Physiol Biochem. 2018;49:2511‐2520. 30261503 10.1159/000493876

[12] Chen S , Wang M , Yang H , et al. LncRNA TUG1 sponges microRNA‐9 to promote neurons apoptosis by up‐regulated Bcl2l11 under ischemia. Biochem Biophys Res Comm. 2017;485:167‐173. 28202414 10.1016/j.bbrc.2017.02.043

[13] Mercer TR , Dinger ME , Mattick JS . Long non‐coding RNAs: insights into functions. Nat Rev Genet. 2009;10:155‐159. 19188922 10.1038/nrg2521

[14] Ebert MS , Neilson JR , Sharp PA . MicroRNA sponges: competitive inhibitors of small RNAs in mammalian cells. Nat Methods. 2007;4:721‐726. 17694064 10.1038/nmeth1079 PMC3857099

[15] Ma F , Wang SH , Cai Q , et al. Long non‐coding RNA TUG1 promotes cell proliferation and metastasis by negatively regulating miR‐300 in gallbladder carcinoma. Biomed Pharmacother. 2017;88:863‐869. 28178615 10.1016/j.biopha.2017.01.150

[16] Duan LJ , Ding M , Hou LJ , Cui YT , Li CJ , Yu DM . Long noncoding RNA TUG1 alleviates extracellular matrix accumulation via mediating microRNA‐377 targeting of PPARgamma in diabetic nephropathy. Biochem Biophys Res Comm. 2017;484:598‐604. 28137588 10.1016/j.bbrc.2017.01.145

[17] Papadopoulos MC , Verkman AS . Aquaporin water channels in the nervous system. Nat Rev Neurosci. 2013;14:265‐277. 23481483 10.1038/nrn3468 PMC3732112

[18] Manley GT , Fujimura M , Ma T , et al. Aquaporin‐4 deletion in mice reduces brain edema after acute water intoxication and ischemic stroke. Nat Med. 2000;6:159‐163. 10655103 10.1038/72256

[19] Yao X , Derugin N , Manley GT , Verkman AS . Reduced brain edema and infarct volume in aquaporin‐4 deficient mice after transient focal cerebral ischemia. Neurosci Lett. 2015;584:368‐372. 25449874 10.1016/j.neulet.2014.10.040 PMC4737527

[20] Zheng Y , Pan C , Chen M , Pei A , Xie L , Zhu S . miR29a ameliorates ischemic injury of astrocytes in vitro by targeting the water channel protein aquaporin 4. Oncol Rep. 2019;41(3):1707‐1717. 30628716 10.3892/or.2019.6961 PMC6365700

[21] Zheng Y , Wang L , Chen M , Pei A , Xie L , Zhu S . Upregulation of miR‐130b protects against cerebral ischemic injury by targeting water channel protein aquaporin 4 (AQP4). Am J Transl Res. 2017;9:3452‐3461. 28804561 PMC5527259

[22] Guizzetti M , Costa P , Peters J , Costa LG . Acetylcholine as a mitogen: muscarinic receptor‐mediated proliferation of rat astrocytes and human astrocytoma cells. Eur J Pharmacol. 1996;297:265‐273. 8666059 10.1016/0014-2999(95)00746-6

[23] Bederson JB , Pitts LH , Tsuji M , Nishimura MC , Davis RL , Bartkowski H . Rat middle cerebral artery occlusion: evaluation of the model and development of a neurologic examination. Stroke. 1986;17:472‐476. 3715945 10.1161/01.str.17.3.472

[24] Li T , Chen Y , Zhang J , Liu S . LncRNA TUG1 promotes cells proliferation and inhibits cells apoptosis through regulating AURKA in epithelial ovarian cancer cells. Medicine. 2018;97:e12131. 30200102 10.1097/MD.0000000000012131 PMC6133603

[25] Wang J , Cao B , Han D , Sun M , Feng J . Long non‐coding RNA H19 induces cerebral ischemia reperfusion injury via activation of autophagy. Aging Dis. 2017;8:71‐84. 28203482 10.14336/AD.2016.0530 PMC5287389

[26] Nielsen S , Nagelhus EA , Amiry‐Moghaddam M , Bourque C , Agre P , Ottersen OP . Specialized membrane domains for water transport in glial cells: high‐resolution immunogold cytochemistry of aquaporin‐4 in rat brain. J Neurosci. 1997;17:171‐180. 8987746 10.1523/JNEUROSCI.17-01-00171.1997 PMC6793699

[27] Li Y , Xu QQ , Shan CS , Shi YH , Wang Y , Zheng GQ . Combined use of emodin and ginsenoside Rb1 exerts synergistic neuroprotection in cerebral ischemia/reperfusion rats. Front Pharmacol. 2018;9:943. 30233364 10.3389/fphar.2018.00943 PMC6127650

[28] Papadopoulos MC , Verkman AS . Aquaporin‐4 and brain edema. Pediatr Nephrol. 2007;22:778‐784. 17347837 10.1007/s00467-006-0411-0 PMC6904420

[29] Thomson DW , Dinger ME . Endogenous microRNA sponges: evidence and controversy. Nat Rev Genet. 2016;17:272‐283. 27040487 10.1038/nrg.2016.20

[30] Zhao L , Sun H , Kong H , Chen Z , Chen B , Zhou M . The Lncrna‐TUG1/EZH2 axis promotes pancreatic cancer cell proliferation, migration and EMT phenotype formation through sponging Mir‐382. Cell Physiol Biochem. 2017;42:2145‐2158. 28813705 10.1159/000479990

[31] Lu Y , Tang L , Zhang Z , et al. Long noncoding RNA TUG1/miR‐29c axis affects cell proliferation, invasion, and migration in human pancreatic cancer. Dis Markers. 2018;2018:6857042. 30595764 10.1155/2018/6857042 PMC6282130

