# Supplementary material for: Long noncoding RNA TUG1 contributes to cerebral ischaemia/reperfusion injury by sponging mir‐145 to up‐regulate AQP4 expression
Source: J Cell Mol Med. 2019 Nov 11;24(1):250–9. doi: 10.1111/jcmm.14712 (PMC6933375; doi:10.1111/jcmm.14712)
Supplement: Supplementary file 1 [file JCMM-24-250-s001.docx]

Figure S1. Knockdown of AQP4 inhibits cell apoptosis, LDH leakage decreases and cell health increases in OGD/R-treated MA-C cells.

A. Astrocytes were visualized by GFAP immunostaining (red). Nuclei were counterstained with DAPI.

B. The morphological changes of MA-C cells under control or OGD/RX condition.

C. QRT-PCR analysis of LncRNA TUG1 expression after TUG1 knockdown.

D. AQP4 levels were detected in 6-h OGD/24-h reoxygenation treatments by Western blotting and quantified.

E. *AQP4* expression was detected in 6-h OGD/24-h reoxygenation treatments using qRT-PCR.

F-G. Cell health and LDH were examined by cell health and LDH assays. The cells were transfected with siAQP4 in 6-h OGD/24-h reoxygenation treatments.

H. Flow cytometry analysis of the cell apoptosis after transfection with siAQP4 in 6-h OGD/24-h reoxygenation treatments.

I. Western blot analysis of AQP4 protein after transfection with siAQP4.

J. QRT-PCR analysis of MIR-145 expression after transfection of an mir-145 inhibitor. siRNA.

siRNA, short interfering RNA; TUG1, taurine-up-regulated gene 1; AQP4, aquaporin 4; qRT-PCR, quantitative real-time reverse-transcription polymerase chain reaction; OGD/R, oxygen-glucose deprivation and reperfusion; GFAP, glial fibrillary acidic protein; DAPI, 2-(4-amidinophenyl)-1H-indole-6-carboxamidine
