# Supplementary figures and images for: Long noncoding RNA TUG1 contributes to cerebral ischaemia/reperfusion injury by sponging mir‐145 to up‐regulate AQP4 expression
Source: J Cell Mol Med. 2019 Nov 11;24(1):250–9. doi: 10.1111/jcmm.14712 (PMC6933375; doi:10.1111/jcmm.14712)

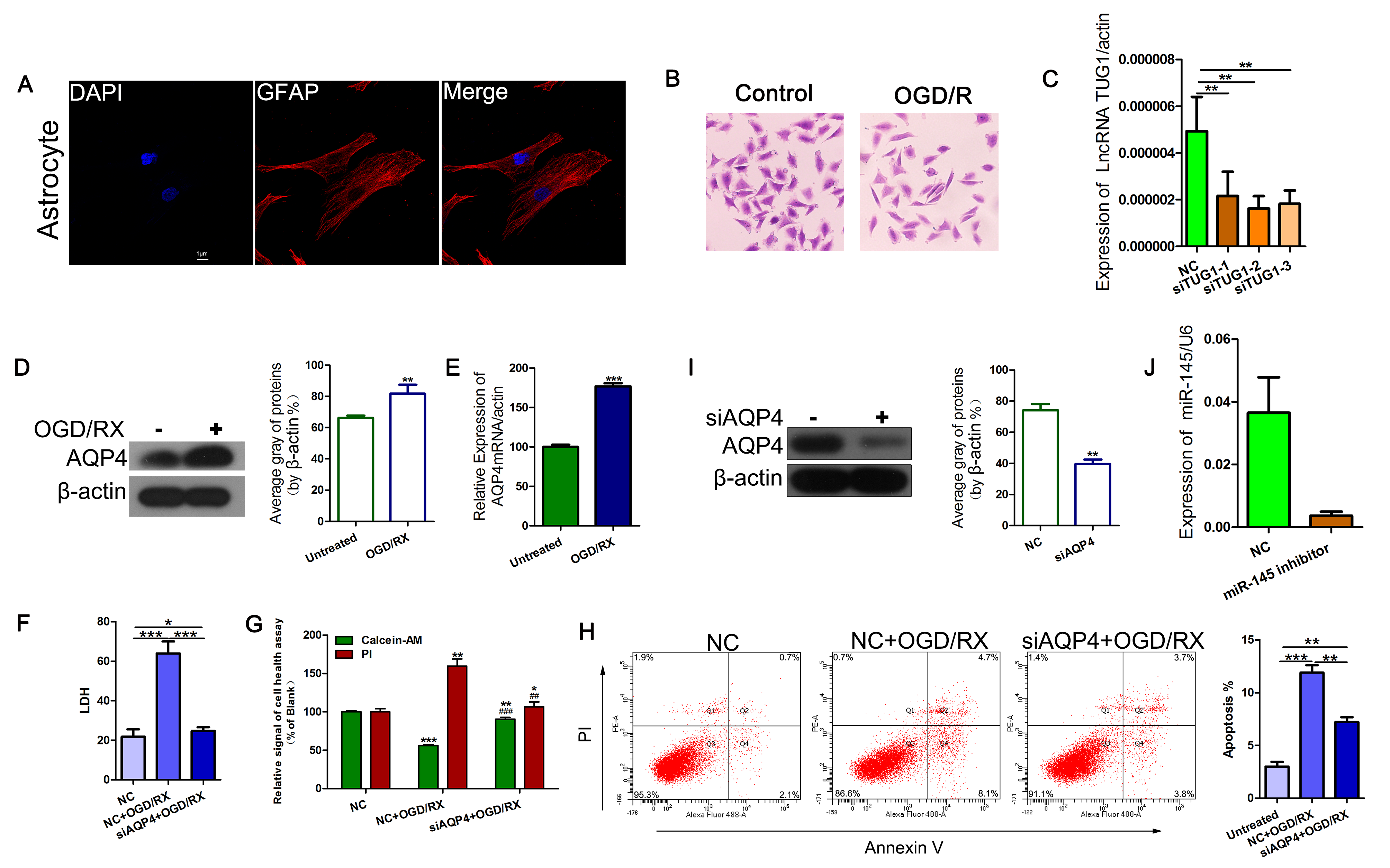

Supplement: Supplementary file 2 [file JCMM-24-250-s002.tif]
